# Parent-Mediated Autism Intervention Through a Culturally Informed Lens: Parents Taking Action and Pivotal Response Training with Latine Families

**DOI:** 10.3390/healthcare12232381

**Published:** 2024-11-27

**Authors:** Kristina Lopez

**Affiliations:** School of Social Work, Arizona State University, Phoenix, AZ 85004, USA; klopez27@asu.edu

**Keywords:** autism, Latine, families, intervention, culturally informed

## Abstract

**Background/Objectives**: The prevalence of autism has increased substantially among Latine children; however, few service systems are prepared to provide culturally relevant services. Parents Taking Action (PTA) is a culturally informed parent-mediated psychoeducation program designed to meet the informational needs of Latine families with children with autism. The purpose of this study was to pilot a hybrid model of intervention by including direct parent coaching through pivotal response training (PRT) along with PTA among Latina mothers of children with autism. **Methods**: Ten Latina mothers of children eight years of age with autism participated in this study. The mothers received PTA from two bilingual trained promotoras (community health workers). PTA provided 14 2 h psychoeducation sessions. The mothers also received four one-hour sessions of PRT from bilingual/bicultural coaches. **Results**: Paired samples t-tests indicated significant increases in the families’ outcomes, their self-efficacy in using the intervention strategies, and their frequency of using strategies, from pre- to post-test. **Conclusions**: This study suggests that culturally informed parent-mediated autism intervention coupled with parent coaching positively affects family outcomes among Latine families of children with autism.

## 1. Introduction

Autism prevalence has multiplied exponentially, affecting 1 in 36 children or 0.8% of the population [1]. Improved identification of autism has occurred among 4- and 8-year-old non-white children [1,2,3]. In 2020, for the first time since the Centers for Disease Control (CDC) collected prevalence data, more 8-year-old Black, Latine, and Asian or Pacific Islander children were identified with autism than white children. While improved identification of autism is necessary, early diagnosis is also critical for optimizing support to individuals with autism and their families [4]. Despite the increase in prevalence, Latine children continue to be diagnosed later than non-Latine white children [5,6]. Latine children more often receive a diagnosis of speech impairment over autism when intellectual disability is also present [7]. Many Latine children diagnosed with autism receive inadequate quality of care and have unmet service needs [6,8,9].

Various intersecting sociocultural factors at the child, parent, provider, community, and policy levels create and enhance barriers to autism diagnostic and treatment services among Latine children [10]. Barriers to early diagnosis and treatment at the family level include immigration status, initial diagnosis rejection among caregivers, health literacy, English fluency, and maternal education levels [10,11,12]. System-level barriers include provider bias and training, abrupt service cancelation, incomplete screening tests, inadequate referrals, and waiting lists [10,11,13]. The impact of these barriers to diagnosis and treatment access is significant given the importance of early intervention for the children’s development, their long-term quality of life, and parental stress [14]. Researchers have underscored the importance of including culture, innovation, and exploratory methods when developing and implementing interventions for culturally and linguistically diverse families [15,16]. Parents Taking Action (PTA) is a unique and culturally informed psychoeducation program developed with the needs of Latine families raising children with autism integrated throughout the curriculum and program delivery. PTA has been shown to meet the informational needs of Latine families of children with autism, enhance parents’ skills to work on their children’s social communication, and enhance parents’ ability to advocate for treatment services [6].

However, PTA is a didactic program that does not include the direct practice of strategies with children and parents. Thus, an objective of this study was to expand the parameters of the PTA program by including direct coaching with parents and children through a form of evidenced-based practice, pivotal response training (PRT). PRT is a form of applied behavior analysis targeting functional and social communication skills by utilizing the natural environment to motivate children with autism [16]. Given the flexibility of PRT, it aligns with core dimensions of PTA, which include flexibility, collaborative goal planning with parents, and the inclusion of the child’s natural environment. Further, the evidence-based communication, social, and behavior management strategies taught in PTA are also featured in PRT, allowing for streamlined practice. The objective of this study was to pilot a PTA and PRT intervention targeting the use of evidence-based social communication intervention strategies among Latine families of children with autism.

### 1.1. Latine Children with Autism

Although the Latine population comprises the largest ethnic minority group in the US, Latine children continue to experience overwhelming disparities in access to health and mental health care [17]. A growing area of concern in mental health for Latine children is the increase in the diagnosis of developmental disabilities, particularly autism diagnoses [18]. The greater autism identification of Latine children has been made possible, yet the persistent disparities in prevalence, age of diagnoses, and treatment access among Latine children are alarming. Between 2002 and 2008, the prevalence of autism among Latine children jumped 110% [19]. The prevalence rate among Latine children continued to increase, exceeding that of white children by 2020 [20]. Yet, Latine children are diagnosed up to two years later than non-Latine white children, even when there is no difference in the children’s age at which their parents report symptoms to their child’s doctor [12]. Factors across the child, parent, provider, practice, and policy levels impact their access to diagnostic and treatment services [8,15,21]. Attempts to alleviate the age of diagnosis disparity among Latine children have included national awareness campaigns, community outreach programs, recommendations for early screening by all pediatricians during baby and child visits, as well as increasing the availability of Spanish language information about autism [18]. Elevated public awareness of autism and improved autism identification has escalated the critical need for quality treatment for Latine children who are diagnosed with autism. Yet, many Latine parents of children with autism continue to report poor access to and quality of treatment services for their children [22,23]. Latine parents have highlighted that they feel uninformed about their child’s diagnosis by providers and disregarded in treatment planning [23]. The lack of providers who are familiar with the cultural and linguistic backgrounds of Latine families multiplies the disadvantage Latine children experience in attempts to access diagnosis and treatment services [10,15,24]. Delays in diagnosis, treatment access, and quality of care early in a child and their family’s autism journey can lead to a lifelong trajectory of adverse care experiences and poorer health and mental health outcomes [9,25]. Suggestions to improve the quality of care and better support families include an increase in the availability of culturally informed practitioners and intervention programs, collaboration between parents and providers in treatment planning, and parent training [26]. The use of parent-mediated interventions is essential to boost the exposure Latine children have to treatment that they may not otherwise receive [15,27]. Astoundingly, few intervention programs have been designed and explored for use with Latine children with autism and their families.

### 1.2. Parents Taking Action

Parents Taking Action (PTA) is a culturally informed intervention program for Latine families. PTA has been effective in enhancing parent outcomes and improving the use of services for Latine children with autism [28,29,30]. PTA comprises 14 2 h sessions, delivered in the homes of Latine families of children with autism by promotoras, or community health workers who are Latina mothers of children with autism. The content of the sessions includes information about autism, developmental milestones, advocacy in educational and other service systems, maternal self-care, and evidenced-based strategies to enhance children’s social communication skills. PTA was developed on the foundation of the ecological validity model [31]. The ecological validity model outlines eight domains including language, persons, metaphors, content, concepts, goals, methods, and context, which must be carefully aligned with the cultural group for which an intervention is developed or adapted. PTA is offered in the language (Spanish or English) preferred by the participants, which comprises the language domain. The person’s domain is incorporated with the implementation of a promotora de salud (community health worker) model, whereby Latina mothers of children with autism or other developmental disabilities from the same community act as participants who are trained to deliver the PTA program. The promotora model thus allows for cultural and experiential matching between participants and promotoras. Metaphors used in the PTA curriculum include cultural sayings or dichos. The content and concept domains comprise cultural values such as personalismo (emphasis on relationships) built into PTA through the evolving dynamic between the promotora and the participant and the use of novelas (dramatic radio dramas) that center on relationships and family. Collaborative goal development is emphasized throughout PTA to address the goals domain. Finally, the methods domain was addressed with a home visit model to mitigate the transportation challenges for families and to create a comforting environment, which can support immigrant families who may have fears of deportation. A randomized control trial of PTA found Latina mothers who received PTA had positive changes in their confidence of and frequency in the use of evidence-based (EB) strategies. The children in this group experienced positive changes in their social communication and the number of services they received from baseline to post-intervention [29]. A follow-up study revealed that the gains in maternal confidence and service receipt were maintained from post-intervention to a four-month follow-up time point for those in the intervention group [30]. These and other research using PTA illustrate the value of this program to mitigate the disparities experienced by Latine children with autism and their families.

### 1.3. Pivotal Response Training

As mentioned above, PTA is a fully didactic program. While video examples are used to demonstrate EB strategies, parents do not work directly with their children and a provider to understand and practice using the strategies in their daily lives. Previous participants in PTA studies have suggested that behavioral coaching with their children during PTA would enhance their understanding of how to use the evidence-based strategies. In this study, we attempted to address this area of need by pairing PTA with pivotal response training (PRT). PRT is an evidence-based naturalistic developmental behavioral intervention embedded with principles of applied behavioral analysis [13]. In PRT, the “pivotal” or core areas impacted by autism, such as motivation and communication, are targeted. By enhancing motivation within the natural environment, PRT provides an opportunity to impact a broad set of functional and social communication skills [13]. PRT was selected as the behavioral coaching model to pair with PTA given the emphasis on using naturalistic opportunities to enhance children’s functional skills and communication. According to Dennison et al. [32] home-supported applied behavior analysis therapies must be inclusive of culturally appropriate targets and practices when working with culturally and linguistically diverse families. However, other research has indicated that early interventionists have avoided providing coaching to parents from cultural or linguistic minority groups, leaving their children with autism at a further disadvantage from receiving the benefits of services [33,34]. Thus, it was imperative that the behavioral coaching model selected for this study allowed for cultural preferences to be included and capitalized on to support parent learning and children’s development. PRT has been identified as an evidenced-based form of parent-mediated intervention [35]. PRT has been tested and used with a variety of children and families from diverse racial, ethnic, and socioeconomic backgrounds [36,37]. For instance, McGarry et al. [37] conducted a study testing the feasibility of online PRT with parents of children with autism who were primarily racial/ethnic minorities. Eleven families completed the entire PRT program and had positive outcomes. Specifically, they learned and utilized the PRT strategies with their children. The children’s social communication was also improved from pre- to post-intervention. Thus, this study demonstrated that PRT can positively impact the parent and child outcomes among racial/ethnic minority populations. Another nuance of the PRT model is the flexibility of implementation that has been found in the research among diverse populations. Dahiya et al. [38] provided a one-day PRT workshop to a diverse sample of parents in rural communities. They found the short training to increase parent confidence in implementing PRT strategies and reduce parental stress. Schuck et al. [39] used telehealth to implement PRT among a diverse sample of children with autism and their parents. The findings demonstrated increases in the amount of words the children produced, in their communication, and in their receptive language scores post-intervention. These studies illustrate the flexibility of PRT implementation.

While there are no PRT studies focused primarily on the Latine population, studies have included a limited number of Latine children and parents [36,38,39]. Only one study has reported the results of PRT among a Latine subsample [36]. More specifically, Baker-Ericzén et al. [36] used PRT among a sample of 158 children between 24 and 113 months of age. Thirty-five percent of the sample was Latine. The parents in the study received a 12-week parent education program that included PRT. The Latine children improved in their communication, daily living, socialization, and adaptive behavior composite from pre- to post-intervention, demonstrating the utility of PRT with Latine children and parents.

### 1.4. The Present Study

By including PRT with PTA, this study provides insight into culturally informed intervention along with the first use of PRT among a completely Latine sample of autistic children and mothers. This article is a revised and expanded version of a paper entitled “Combining Parents Taking Action and pivotal response training to support Latinx mothers of children with ASD”, which was presented at the International Society for Autism Research Annual Meeting Annual Conference’s virtual meeting on 3 June 2020 [40].

### 1.5. Research Question and Hypotheses

1. Do the Latina mothers who receive the PTA/PRT intervention show improvements in their family outcomes post-PTA/PRT intervention?
  - We expect that the Latina mothers who participate in the PTA/PRT intervention will show significant improvements in their family outcomes post-intervention.
2. Do Latina mothers improve their efficacy in using evidence-based strategies to improve their children’s social communication post-PTA/PRT intervention?
  - We expect that the Latina mothers who participate in the PTA/PRT intervention will show significant improvement in their efficacy of using evidence-based strategies post-PTA/PRT intervention.
3. Do Latina mothers increase their frequency of using evidence-based strategies to improve their children’s social communication post-PTA/PRT intervention?
  - We expect that the Latina mothers who participate in the PTA/PRT intervention will show significant increases in their frequency of using evidence-based strategies to improve their children’s social communication post-PTA/PRT intervention.

## 2. Materials and Methods

Given the pilot nature of this study, all the eligible participants were offered the program after meeting the study eligibility. The mothers received 14 2 h sessions of the PTA program in their homes from two promotoras (community health care workers). The PTA content covers general information about autism, treatment services, advocacy, self-care, and strategies to enhance children’s social communication. PTA is a didactic program that requires mothers to follow a participant manual, while promotoras utilize a promotora manual to guide them through the informational material. Video examples and audio materials are also included as adult teaching strategies. Promotoras provide participants with a supplementary resource guide about local autism and general resources for evidence-based treatment, as well as guides for navigating services. If a parent needs additional supports not found in the guide, the promotora communicates with the study director who coordinates with social work interns to identify possible external resources.

The mothers also received 4 one-hour sessions of pivotal response training (PRT) from bilingual/bicultural coaches. The PRT coaches were undergraduate and graduate students who completed training in PRT from a certified PRT trainer at a local autism service organization. The trainer provided feedback to the coaches before they delivered PRT to the mothers in the study. The one-hour PRT sessions were delivered immediately after four corresponding PTA sessions that focused on teaching mothers evidence-based skills to work with their children on social communication and behavior management (i.e., Play Together, Learn Together Creating Everyday Opportunities to Encourage Communication; Challenging Behavior is Communication; Making Challenging Behaviors Less Likely and Responding Appropriately When They Do Happen). The promotoras and PRT providers interacted to confirm the information about meeting with the mothers. However, the study coordinator served as a liaison to avoid confusion about the ease of communication. The skills-based sessions of PTA were session 7, 8, 10, and 11. Once a promotora completed session 7, they would contact the study coordinator to debrief. Promotoras would also inform the coordinator about the best time/day for the mother to meet. The study coordinator would then contact the PRT provider to let them know it was time to schedule the mother for a PRT session.

All the intervention materials were offered in Spanish and English. The mothers were asked to choose which language they preferred to receive the intervention. Of the ten who started the intervention, four requested the entire program be delivered in Spanish. The remaining mothers requested the program in English. The intervention package was delivered in the homes of the mothers. The children were only required to be present for the PRT sessions.

The mothers completed questionnaires regarding their child, parent, and family outcomes pre-intervention, post-intervention after completing all the PTA/PRT intervention sessions and follow-up. Ten minutes of parent–child interaction were recorded in audio–visual form at these time points if the mothers agreed to the taping. After all the participants completed this, the intervention focus groups were conducted in Spanish and English to assess for the feasibility of the intervention. Four mothers participated in the focus groups (two in Spanish, two in English).

### 2.1. Participants

Participants were recruited from a metropolitan area of the Southwest region in the United States. According to Liu et al. [41], Latine children with autism in metropolitan areas tend to have the access to the fewest resources per child compared to their white counterparts. During the recruitment year of the present study (2018), the population of the area was 4,307,033 [42]. The demographics were 55.7% white (non-Hispanic), 21.9% white (Hispanic), 1.31% multiracial (Hispanic), 2.31% multiracial (non-Hispanic), 6.82% other (Hispanic), 5.18% Black or African American (non-Hispanic), 4.06% Asian (non-Hispanic), and 1.59% American Indian and Alaskan Native (non-Hispanic) [43]. Approximately 91.1% of the population were US citizens. The median household income in 2018–2022 was USD 80,675 in the county [42]. In 2018, the median household income was USD 62,000 [43]. The percentage of children living in poverty was 21.5 in 2018 [43].

Participants were recruited through local Spanish language support groups for families of children with autism and/or other developmental disabilities, autism service agencies, and autism support groups. Recruitment was conducted by presenting the study information in groups, on Facebook, and via word of mouth. Thirteen inquiries were initially received. However, two inquiries were from families outside of the geographic area to participate. A third inquiry was from a family that did not want to participate after hearing more about the study and indicating they were maxed out on time with the number of services they were already receiving, and the mother could not participate. Ultimately, ten Latina mothers of children diagnosed with autism met the criteria for participation and agreed to be in the study. To participate, the mothers were required to identify as Latina and have a child diagnosed with or at-risk of autism. The mothers were not required to be immigrants. The children were required to be 8 years of age or under. The individuals who did not meet these criteria were not included in the study.

The mothers were offered $20 per assessment (baseline, post-intervention, follow-up, focus group) for their participation. A previously established advisory board comprising autism, cultural, parental, and community stakeholders provided recruitment method suggestions. Due to the attrition of three mothers, this study includes data from the seven mothers who completed all the study components. The majority (71.4%) of the children were male. The children ranged from 2 to 7 years of age. Although the children were not required to be formally diagnosed with autism, all the children were previously diagnosed with autism by a health professional per parent report. Parent reports of autism have been found to have high reliability [44]. Other demographic information is available in Table 1.

### 2.2. Measures

A demographic and health questionnaire regarding the child (i.e., age, age of diagnosis, etc.) and mother (i.e., age, annual income, employment, education, general health, etc.) was completed during the baseline visit.

The Childhood Autism Rating Scale, 2nd edition (CARS2-ST; [45]) was utilized at the baseline assessment to determine the autism severity among the children in the study. The CARS2-ST also informally confirmed the parent-reported autism diagnosis the children had previously received from a health professional. The CARS2-ST is a 15-item autism assessment tool designed to be used following the direct observation of a child. In this study, the children were observed for 15 min, after which a trained researcher completed the assessment tool. Each item was rated between (1) within the normal limits for that age to (4) severely abnormal for that age. The raw scores were then summed for a total score. The cutoff scores for autism vary by age. However, the overall cutoff score is 28. Scores can be further categorized into three severity groups: “minimal-to-no symptoms”, “mild-to-moderate symptoms”, and “severe symptoms”. The CARS2-ST has been used across a wide range of children. A recent systematic review and meta-analysis assessing the accuracy of the CARS and the Diagnostic and Statistical Manual of Mental Disorders, Fifth Edition resulted in sensitivity of 0.86 and 0.71 and specificity of 0.79 and 0.75, respectively [46].

The Social Communication Questionnaire (SCQ; [47]) current form was used to determine the children’s degree of social communication skills. The SCQ was utilized at each assessment time point. The participants were offered both the English and previously translated Spanish version to complete. The SCQ includes 40 “yes” or “no” items, such as “Does he/she ever talk with you just to be friendly (rather than to get something)?” or “Does he/she respond positively when another child approaches her/him?”. The total number of Yes responses were summed for a total social communication score. Lower scores indicated less social communication impairment. The mothers were asked to complete this form during the pre-, post-, and follow-up data collection points. The SCQ has demonstrated a range of 0.88–0.90 and a specificity of 0.72–0.86 [48].

The Family Outcome Survey—Revised (FOS-R; [49]) was developed by the Early Childhood Outcomes Center with support from the Office of Special Education Programs of the U.S. Department of Education. The 24-item measure was developed to assess five areas of early intervention program support: (1) understanding your child’s strengths, needs, and abilities, (2) knowing your rights and advocating for your child, (3) helping your child develop and learn, (4) having support systems, and (5) accessing the community. The responses range on a Likert scale from (1) not at all to (5) completely. Sample items include, “We know who to contact and what to do when we have questions or concerns” and “We are able to talk with other families who have a child with similar needs.” The FOS-R has been translated to Spanish previously. The FOS-R was completed at the baseline, post-intervention, and follow-up time points. Previous studies have found Chronbach’s alpha for the subscales of the FOS-R to range from 0.78 to 0.93 [49,50].

The Self-Efficacy of Using Strategies includes eleven items assessing the level of confidence the mothers felt using the evidence-based (EB) strategies they learned during the intervention program. This scale was developed by the original PTA research group and translated to Spanish. The efficacy items were anchored by the phrase, “I feel confident…”, with response options ranging on a Likert scale from (1) completely disagree to (4) completely agree. The sample items included “I feel confident modeling for my child what I want him/her to do” and “I feel confident using social stories”. The responses were summed for a total efficacy in use of strategies score.

A Frequency of Using Strategies test was used to determine the frequency mothers utilized the EB strategies; the mothers were asked how often they used the 14 EB strategies. As with the efficacy scale, this measure was developed by the original PTA research group and translated to Spanish. The sample items included, “How often have you tried to make learning fun by incorporating your child’s favorite materials, activities, and interests?” and “How often have you used a token economy to reward appropriate behavior?”. The response options for the 14 EB strategies ranged on a Likert scale from (1) Never to (4) Always. To calculate the total frequency of the Using Strategies score, all the responses were added, with the greater scores indicating more frequency of use.

### 2.3. Data Analysis

The subscales for each dependent variable were calculated and the data were analyzed using SPSS 28. A z-test was applied as a normality test, using skewness and kurtosis for the variables. The data were normally distributed for all but two outcomes (community access and EB services) and no missing data were observed for the variables of interest included in the present study. For the variables meeting assumptions of normality, paired sample *t*-tests were completed to compare the baseline and post-intervention scores for the changes in family outcomes, the mothers’ efficacy in using the strategies, the frequency of using the strategies, and the child outcomes among the seven mother–child dyads that had complete data for both timepoints. For the outcomes that were non-parametric (community access and EB services), Wilcoxon Signed-Rank tests were conducted (Table 3). The level of significance for all the *p* values was below 0.05. *Cohen’s d* was calculated using the G*Power tool version 3.1.9.7 in Windows to account for the within-subjects design [51]. The intervals for the classifying effect sizes were small (*d* = 0.2), medium (*d* = 0.5), and large (*d* ≥ 0.8) [52].

## 3. Results

### 3.1. Parent Variables

The paired samples t-tests showed significant differences in three family outcomes pre- vs. post-intervention for understanding their child’s strengths, needs, and abilities (10.29 ± 3.20 vs. 13.00 ± 2.31, *p* < 0.05, d = 1.26), knowing their rights and advocating for their children (10.00 ± 5.20 vs. 17.29 ± 2.81, *p* < 0.05, d = 1.51), having a support system (11.43 ± 5.26 vs. 17.86 ± 2.41, *p* < 0.05, d = 1.37), and the total family outcome scores (48.00 ± 16.02 vs. 65.14 ± 7.24, *p* < 0.05, d = 1.14) (Table 2). Paired samples *t*-tests indicated that the mothers’ report of efficacy using the EB strategies differed significantly pre- vs. post-intervention (34.43 ± 3.21 vs. 42.14 ± 3.24, *p* < 0.05, d = 1.52). The frequency for which the mothers used the EB strategies was also significantly different pre- vs. post-intervention (36.57 ± 3.41 vs. 46.29 ± 4.61, *p* < 0.05, d = 1.59) (Table 2). The effect size for each of these significant results was large.

### 3.2. Child Variables

No significant differences were found for either of the social communication scores, the restrictive and repetitive behaviors, the total SCQ scores, or the number of DD or EB services the children received pre- vs. post-intervention. See Table 2 and Table 3 for statistical results.

## 4. Discussion

The intersection of the growth of the Latine population and the amplified prevalence of autism has heightened attention on the persistent disparities in the diagnosis and treatment of Latine children with autism. Researchers have called for the development and implementation of exploratory and innovative interventions to meet the needs of culturally diverse families [27,31]. The present study answered the call by investigating a culturally informed psychoeducation program, PTA, for the Latine parents of children with autism and extended the impact by combining it with PRT, an evidence-based intervention based on applied behavior analysis. Prior studies of PTA have found that parents desired direct coaching and feedback on the use of EB strategies with their children in addition to the original didactic design of the PTA program [28]. In addition, previous research indicates that Latine families are likely to not receive coaching from early intervention service providers based on cultural and linguistic factors as well as other provider-perceived barriers [19]. After carefully considering multiple models, PRT offered a fitting model of evidence-based practice for several reasons. First, PRT has been identified as a parent training intervention for the parents of children with autism [35]. Second, PRT has previously been used with racial/ethnic diverse families [36,37]. Third, PRT includes similar methods and strategies to improve children’s social communication as found in PTA, such as utilizing the natural environment, utilizing everyday routines for skills practice, and engaging parents in collaborative goal planning [35]. Thus, the PTA/PRT intervention tested in this study combined two previously established evidence-based interventions to determine whether they could meet the informational and skill development needs of Latine parents of children with autism.

Ten Latina mothers and their children with autism participated in this study. All the families were provided with the PTA/PRT intervention in their homes. Two bilingual, Latina mothers of children with developmental disabilities were trained as promotoras to deliver the PTA program. Two bilingual/bicultural Latina graduate students were trained to deliver PRT. The parent and child assessments were conducted at baseline, post-intervention, and in the follow-up. Seven mothers completed the entire intervention protocol and all three of the assessments. Four mothers participated in focus groups about the feasibility of the intervention. Two mothers left the study due to other family needs that interfered with their ability to complete the intervention or the program fit. One mother reported that there were too many interventions her child was in to keep track of. Another mother stated that she did not want to complete more forms. The third mother decided the intervention was not an appropriate fit for her needs after completing several sessions of PTA.

The study findings indicated that after receiving the intervention, compared to baseline, the mothers had significant increases in their understanding of their children’s strengths, needs, and abilities, and the knowledge of their rights and how to advocate for their children, as well as an improved understanding of their support system. Lastly, the family outcomes significantly increased from baseline to post-intervention. Increased family outcomes are linked to greater buy-in effectiveness of parent-mediated interventions in community settings [53]. These findings suggest that the PTA/PRT intervention has the potential to fill the informational gap for Latine families raising children with autism. These results mimic previous research finding that PTA positively impacted family outcomes from pre- to pos-t-intervention [29,54].

These findings indicated that the PTA/PRT intervention had a positive impact on the Latina mothers’ efficacy in using the evidence-based intervention strategies with their children from pre- to post-intervention. Parental self-efficacy has been identified as an essential element in parent-mediated intervention for children with autism given the correlation it has to parent stress, family outcomes, the degree to which parents are involved in their children’s intervention, and parents’ ability to be successful with the implementation of intervention strategies [55]. Thus, the efficacy changes in the present study have broader implications for the impact of the PTA/PRT intervention. For instance, the parents of children with autism who have increases in their self-efficacy are more involved in their children’s therapy and are more satisfied with their treatment [56]. Lastly, it is possible that enhancing mothers’ efficacy may have implications for mothers’ ability to advocate for their children [21].

Lastly, the study findings demonstrated that the mothers increased the frequency of using the evidence-based strategies from pre- to post-intervention. Both the PTA’s and PRT’s principles support parents’ use of evidence-based strategies by encouraging them to implement them in everyday settings with their children. According to Kashinath et al. [57], parents who embed strategies into their daily routines are likely to use them more often and find them useful to support their children’s social communication. The self-reported increases in the efficacy and frequency of using the evidenced-based strategies that the mothers reported suggests that the mothers were active participants in the intervention, learned important skills to enhance their children’s social communication, and were willing to use them with their children after receiving the PTA/PRT intervention. Thus, not only did this study offer a unique, culturally informed model of intervention that fills the informational gaps of Latine families raising children with autism, but also enhanced their efficacy and skill development needs to support their children’s social communication with evidence-based strategies.

The social validity of the intervention was determined by the solicitation of feedback about the intervention, including experiences with the promotoras and PRT coaches. The mothers shared positive responses about the intervention including their satisfaction with the location of the intervention, information about autism and advocacy, interactions with the promotoras and PRT coaches, naturalistic strategies featured in the intervention, and flexibility of the program scheduling. The area of dissatisfaction was with the videos featured in the PTA materials. PTA includes audio and videos in select sessions to demonstrate content. However, technical difficulties disrupted the flow of play at times. The mothers indicated frustration with such disruptions and suggested new videos or simply reading the content from the manual instead of trying to watch the video. The mothers also reported that it would have been helpful to streamline the PTA and PRT content within the manual. This information is especially helpful to the research team as they work to enhance the intervention manuals for future use. Overall, the mothers emphasized that they and their children benefited from the PTA/PRT intervention and would recommend the program to other Latine families.

### 4.1. Limitations

There are several limitations to this promising pilot study that require discussion. The generalizability of this study is limited by its small sample size. However, the effect sizes of the results indicate a large effect that holds statistical and clinical significance. The convenience sample presents selection bias, given that the participants were families connected in some way to agencies serving children with autism and/or autism support groups. Thus, the results may reflect outcomes for families who are in a particular phase of acceptance of their child’s diagnosis and ready to engage in treatment. The absence of a control group does not allow for specific determinations about whether it was the intervention itself that led to the changes in the baseline to post-intervention ratings across the various parent and family outcomes. The children’s diagnosis was based on the parent’s reports of previous diagnosis from a health professional. To address this possible limitation in reporting, the CARS2-ST was conducted with each child by a trained researcher. The CARS2-ST scores confirmed the diagnosis among all the children and provided a level of severity for each child’s autism. Additionally, parent reports of autism has been identified as highly reliable sources [44]. The heterogeneous clinical presentation of the child participants based on the CARS2-ST presents a limitation. One child was assessed as severe, whereas all the others were assessed as mild to moderate. Although the promotoras and PRT coaches were able to adapt to the child’s severity in sessions, the children and mothers may have required more/fewer sessions based on the child’s severity, which was not addressed. The lack of findings for children as measured by the SCQ current form may have been limited by the psychometrics of the measure itself. Previous studies have identified the current form to be limited in its psychometric capacity to measure change over time [58]. All the parent measures were self-reported, which may have been affected by social desirability. The focus group data may reflect social desirability. An anonymous survey may provide less biased feedback about how the mothers experienced and liked/disliked the intervention program. The parent report nature of the SCQ may also have limited our ability to detect the differences in the children’s social communication. Furthermore, based on the data collection methods and analyses, there is no way to determine which effects reported in this study were specifically linked to PTA vs. PRT. Finally, we focused the present study on the pre- and post-data only. We plan to develop a future paper to develop the qualitative data and analyze the follow-up data, and we suggest other researchers include follow-up measures of the parent and child outcomes in their own work.

### 4.2. Implications for Practice

This study offers several implications for practice with Latine families of children with autism. Given that the delivery mode utilized promotoras de salud, the study demonstrated an opportunity to formally integrate the Latine community into autism practice. The promotora model is a cost-efficient approach to informing Latina mothers about their children’s autism and how to navigate service systems. By providing the promotoras with formal training, their own leadership capacity was developed, enhancing their opportunities for career advancement and their abilities to support their own children. This by-product of the intervention model has been demonstrated in other studies utilizing a promotora de salud model [59]. The positive reception of PRT as the ABA framework by the mothers in the study suggests that PRT is an acceptable evidence-based treatment among Latina mothers. The naturalistic and individualized elements of PRT allowed for it to be embedded within the cultural context of the participants and the PTA content while advancing the use of evidence-based practice. Further, integrating PTA and PRT extended the participants’ opportunity to practice skills to enhance their children’s social communication and receive direct feedback about their skills growth. Although early intervention programs within Part C of IDEA offer coaching elements, a recent study found that many early interventionists report do not provide coaching to linguistic and cultural minority families [36]. Specifically recruiting bilingual/bicultural students to deliver the PRT coaching alleviated several barriers to service delivery that saturated early intervention programs in community settings [36,37]. Combining the promotora model of PTA with PRT policymakers can optimize various treatment reimbursement systems to cover the cost of care.

### 4.3. Implications for School Age and Beyond

The present study offers implications for autistic Latine children throughout their lifespan and families. Recently, PTA has been growing in use with adolescents and the transition to adulthood population. For instance, Garcia Torres et al. [60] adapted PTA for youth aged 10 to 17 and their parents in Bogota, Colombia. To meet the needs of the parents of pre-adolescents and adolescents, the topics of sexuality and adolescence were included in the youth-focused PTA program; Padres Unidos en Transformación y Empoderamiento (PUENTE) was developed based on PTA in San Diego, CA for 11 to 16-year-old Latines with developmental disabilities [61]. In PUENTE, the topics expanded to developmental milestones within peer relationships and independence across daily living skills. The parents were also provided with information about the services aligned for 11 to 16-year-old youth in their regions. Both adaptations of PTA for youth were found to positively impact the outcomes in knowledge about autism, connecting families with community support, and skill mastery among the Latine parents post-intervention, demonstrating the utility of core elements of PTA with Latine adolescents and their families.

### 4.4. Implications for Future Research

Despite the small sample size, this study offers insight for testing the intervention with a larger, more heterogeneous sample of Latine children and families. A randomized control trial of the intervention, including a PTA group, would help to parse the effect of PRT separate from PTA on parent outcomes, and improve causal inferences. Future research should include a heterogeneous Latine sample. Additionally, future directions should seek to include the direct measures of children’s social communication. It is possible that a more precise measure such as weekly recordings of the children may reveal important changes in their social communication. Adding a weekly recording of the mother–child dyad engaging in play activities could reveal more nuanced information about how mothers’ skill set working with their children changes over time. A more objective measure of feasibility or participant satisfaction beyond focus groups would provide valuable information about the feasibility of the intervention. It would be insightful to gather data about the professional development enhancements of the promotoras throughout their employment on intervention and beyond, such as whether the promotoras added more hours of training to their experience, joined support groups, were offered and/or accepted other employment opportunities, etc. The same information could be gathered for those taking on the role of PRT coaches. Such information would be helpful to understand the larger community impact and sustainability of the intervention [57]. The lack of changes in the number of treatment services the children received from pre- to post-intervention may reflect the challenges Latine families encounter as they navigate the Arizona Early Intervention Program and Division of Developmental Disabilities. Rather than a failure of the study intervention, this finding may be indicative of systemic issues in need of change. To determine this, future research will need to compare the findings in autism services and the service access across different geographic areas to examine the state-level and across-state differences. For instance, Zeng et al. [30] found site differences in the services received from pre- to post-intervention, comparing the effects of PTA in states with different autism service delivery systems. The authors suggested that state-level differences may have impacted the degree to which the participants could utilize the advocacy skills learned in PTA to increase the number of services the children were able to receive.

## 5. Conclusions

The present study provided the findings of a pilot intervention study of Parents Taking Action combined with pivotal response training among Latine children with autism and their families. The findings revealed that the seven mothers who received the PTA/PRT intervention had significant increases in understanding their children’s developmental needs, in their knowledge of how to advocate for them, in their understanding of their rights, and in their awareness of their support system after completing the PTA/PRT intervention. The mothers also reported greater efficacy in their use of evidence-based strategies and a greater frequency of using the evidenced-based strategies post-intervention. These findings emphasize the utility of an exploratory and innovative intervention for Latine children with autism and their families. This paper contributes to the knowledge base on family support interventions responsive to the increasingly culturally and linguistically diverse environments in which individuals with intellectual and developmental disabilities, particularly autism, live.

## Figures and Tables

**Table 1:** Sample demographics of participants at baseline.

Baseline Characteristic	*N* ^1^	%
Mother		
Mean age in years (*SD*)	38.57 (8.44)	
Married or living together	6	85.7
Level of education		
Less than high school	1	14.3
High school	2	28.6
Any post-secondary education	4	57.1
Annual household income ^2^		
USD 60,000 or more	1	14.3
USD 30,000–59,999	2	28.6
USD 29,999 or less	1	14.3
missing	3	42.9
Employed part-time	3	42.9
Foreign born	5	71.4
Good/excellent health	6	85.7
Child		
Mean age in years (*SD*)	3.57 (1.62)	
Male	5	71.4
CARS severity		
Minimal to no symptoms	--	
Mild to moderate	6	85.7
Severe	1	14.3

^1^ *N* = 14; mothers *n* = 7, children *n* = 7; ^2^ per the US Census (n.d.), the median household income was USD 80,675. The income below the poverty level was USD 20,780, the income below 150% of the poverty level was USD 31,170, and the income below 200% of the poverty level was USD 41,560 (family of three) (Maricopa County Human Services Department, 2018).

**Table 2:** Paired sample *t*-tests.

	Pre		Post		*t*(6)	*p*	*Cohen’s d*
	*M*	*SD*	*M*	*SD*			
Parent Outcomes							
Understand child’s strengths, needs, and abilities	10.29	3.20	13.00	2.31	−3.36	0.02 *	1.26
Know your rights and advocate for your child	10.00	5.20	17.29	2.81	−3.43	0.01 *	1.51
Help your child develop and learn	10.14	4.60	13.71	1.89	−1.99	0.09	0.75
Have a support system	11.43	5.26	17.86	2.41	−3.63	0.01 *	1.37
Total family outcomes	48.00	16.02	65.14	7.24	−3.00	0.02 *	1.14
Efficacy in use of EB strategies	34.43	3.21	42.14	3.24	−4.04	0.01 *	1.52
Frequency using EB strategies	36.57	3.41	46.29	4.61	−4.21	0.01 *	1.59
Child Outcomes							
SCQ social communication	6.43	2.76	6.29	2.69	0.18	0.86	0.07
SCQ RRBs	4.14	2.12	4.00	2.38	1.14	0.74	0.13
SCQ total	26.57	12.35	23.57	9.16	1.78	0.13	0.68
DD services	2.57	1.90	3.29	1.11	−1.26	0.25	0.48

* Indicates statistically significant change at the 0.05 level.

**Table 3:** Wilcoxon Signed-Rank test results.

	Negative Ranks			Positive Ranks			Test Statistics		
	*n*	Mean Rank	Sum of Ranks	*n*	Mean Rank	Sum of Ranks	Ties	*Z*	*p*
(community access-T2 community access)	7	2.00	4.00	7	4.80	24.00	0	−1.703 ^a^	0.089
(EB services-T2 EB services)	7	0	0	7	1.50	3.00	5	−1.342 ^a^	0.180

^a^ Based on negative ranks.

## Data Availability

The data presented in this study are available on request to the corresponding author per the Institutional Review Board guidelines.
